# Active mitochondria in healthy spiny mouse fibroblasts resemble megamitochondria and remain resilient across lifespan

**DOI:** 10.1101/2025.10.02.680123

**Published:** 2025-10-04

**Authors:** Ebenezer Aryee, Ajoy Aloysius, Sandeep Saxena, Hemendra Vekaria, Patrick G. Sullivan, Ashley W. Seifert

**Affiliations:** 1 Department of Biology, University of Kentucky, Lexington, KY 40508, USA; 2 Aligning Science Across Parkinson’s (ASAP) Collaborative Research Network, Chevy Chase, MD, 20815, USA; 3 Department of Neuroscience, University of Kentucky, Lexington, KY 40506, USA; 4 The Spinal Cord and Brain Injury Research Center (SCoBIRC), University of Kentucky, Lexington, KY 40506, USA; 5 Lexington VA Medical Center, Lexington, KY 40506, USA

**Keywords:** mitochondria, mitochondrial metabolism, electron transport system, reactive oxygen species, regeneration, aging

## Abstract

Although they exhibit limited regenerative ability of some tissues and organs shortly after birth or towards the end of fetal development, humans and laboratory mammals quickly transition to producing scar tissue for tissue repair. In contrast, spiny mice exhibit complex tissue regeneration as adults and provide a blueprint for how regeneration can occur throughout adulthood in mammals. Fibroblasts are key mediators of wound healing outcomes and prior work uncovered that cells from highly regenerative mammals (spiny mice and rabbits) exhibit extreme resistance to oxidative stress compared to those from non-regenerating laboratory mice and rats. Using a battery of cellular tests in primary ear pinna fibroblasts from highly regenerative and non-regenerative mammals, we find that cells from spiny mice and rabbits exhibit a baseline preference for glycolysis supporting a lower ROS-producing basal state. Uniquely, spiny mouse fibroblasts exhibit large, spherical, depolarized mitochondria similar to megamitochondria identified in pathological tissues. The megamitochondria phenotype was present across lifespan in ear pinna fibroblasts from fetal, young and old spiny mice. While rabbit, mouse and rat fibroblasts had polarized tubular mitochondrial networks typical of adult mammalian fibroblasts, isolated rabbit and spiny mice fibroblasts shared lower oxygen consumption efficiency even in the absence of a potential gradient. Taken together, our results support that a shared metabolic signature exists in stromal cells from highly regenerative mammals, although possibly driven by different mechanisms, to converge on a ROS-resistant phenotype which ultimately helps supports tissue regeneration.

## INTRODUCTION

It is widely accepted that most mammals repair damaged skin and musculoskeletal tissues with a scar (aka fibrotic repair). In response to tissue damage, a stereotypical sequence of events is initiated beginning with hemostasis and inflammation (1). Shortly thereafter, keratinocyte migration over the injury site restores barrier function (re-epithelialization) and a provisional extracellular matrix (ECM) is produced by cells in the wound site. Ultimately, this collagen-rich matrix is remodeled into mature scar tissue, and this overlapping sequence of events is the normal outcome of tissue damage in laboratory rodents and humans. In contrast, some vertebrates can functionally reconstruct injured tissues via reparative regeneration. While vertebrate regeneration typically combines processes observed during fibrotic repair and embryonic development (2, 3), key mechanisms that predispose how cells react to injury via regeneration or fibrotic repair remain obscure.

Historically, regeneration among vertebrates has been studied in fish and amphibians using the heart, fins/limbs and tail. Spiny mice (*Acomys spp*.), however, have emerged over the last decade as an important homeothermic model to investigate complex tissue regeneration in mammals (2). The regenerative abilities of *Acomys kempi* and *Acomys percivali* were first discovered as a response to full-thickness injuries to the skin and ear pinna (4). It was observed that instead of scarring, ear-punch injuries in these species resulted in complete restoration of dermis, hair follicles, sweat glands and cartilage. Subsequent work, using captive bred *Acomys dimidiatus*, *A. kempi* and *Oryctolagus cuniculus* (New Zealand white rabbit) showed that ear pinna regeneration was indeed a robust regeneration model (5, 6) reflecting previous work with rabbits (7–10). Further extending this work, broader multi-species comparisons between regenerating (spiny mice, brush-furred mice) and non-regenerating rodents have shown that enhanced regenerative ability is not widespread among rodents (11) and provides a system to identify cellular features required for regeneration.

Appendage regeneration involving complex tissue assemblages requires accumulation of resident cells that proliferate and expand prior to cell differentiation; a transitional period referred to as blastema formation (12, 13). Importantly, what distinguishes cells transiting through a blastema from those that form a tissue analgen during development is that cells in the wounded tissue experience high oxidative stress from reactive oxygen species (ROS) and exposure to inflammatory cells and their products. Collectively, cells strive to maintain tissue and organismal homeostasis in the face of injury, disease and infection. Thus, cells act as frontline sensors to detect and mitigate extrinsic stressors (inflammatory, oxidative, etc.). The sensing capacity of cells depends, in part, on a baseline metabolic state influenced by mitochondrial phenotype which is highly sensitive to deviations in biomolecules between various cell compartments (14). Injury disrupts the local tissue microenvironment by altering oxygen and metabolite concentrations, which in turn affects conditions such as pH and ion flux. These alterations upset cellular equilibrium and activate the cell damage response (CDR), triggering changes in cellular behavior (15, 16). Therefore, cell autonomous features governed by mitochondrial and metabolic states can regulate genomic changes and direct resident cells to carry out a regenerative response (17, 18).

Mitochondria contribute to cellular homeostasis and survival through energy production and biomolecule synthesis, but also control cell state, redox balance and the cellular stress response. To produce energy, mitochondria use glucose, fatty acids and amino acids (mostly glutamine), and the breakdown of glucose and fatty acids results in the production of acetyl coA, a key intermediate that is fed into the TCA cycle. Glutamine enters the TCA cycle as alpha ketoglutarate after glutaminolysis. Beyond providing pyruvate and subsequently acetyl coA for more productive ATP production through the TCA cycle and the electron transport chain (32 ATP in OxPHOS, 2 ATP from glycolysis alone), glucose utilization through glycolysis contributes directly to the synthesis of biomolecules like nucleotides and other biomass building blocks, to support cell proliferation as seen in cancer cells and activated T cells (19).

A connection between metabolic state and cell proliferation emerged from observing the Warburg effect in cancer cells where in the presence of ample amounts of oxygen, glucose breakdown through glycolysis was shifted towards lactate production, avoiding carbon loss through TCA produced CO_2_ (Warburg, 1956). Specifically, halfway through glycolysis, intermediates can be shunted for nucleotide and NADPH synthesis via the pentose phosphate pathway. NADPH synthesized from this pathway is utilized in redox balancing (20) and biomolecule synthesis to support cell proliferation (cholesterol, tetrahydrofolate, proline, fatty acids and deoxyribonucleotides) (21). A similar observation has been noted for cells participating in tissue regeneration (22, 23) suggesting metabolic reprogramming of connective tissue fibroblasts may be a key feature of vertebrate regeneration. Beyond cell proliferation, these distinctive metabolic signatures are equally pronounced in embryonic stem cells (ESCs). Compared to differentiated cells, ESCs are more dependent on glycolysis compared to OxPHOS (24, 25). The correlation between cell potency, proliferation and glycolytic bias becomes more intimately linked in the case of cellular reprogramming, where reprogramming efficiency of fibroblasts to induced pluripotent stem cells (iPSCs) is linked to a pro-glycolytic metabolic state, with efficiency increased and decreased significantly in response to glycolytic inhibitors and augmenters respectively (26, 27).

Fibroblasts mediate the last three stages of wound repair (rev. in 28) and comprise the major proliferative cell type during regeneration (29, 30). This raises the question as to whether cell autonomous mechanisms governed by cellular metabolic state in fibroblasts may regulate how this critical cell type governs the healing response. Compared to mouse (*Mus musculus*) fibroblasts, *Acomys* fibroblasts show increased resistance to TGFβ-induced myofibroblast activation (31). Additionally, *Acomys* and rabbit (*Oryctolagus cuniculus* – another highly regenerative mammal) fibroblasts are highly proliferative and resistant to radiation and ROS-induced senescence compared to fibroblasts obtained from non-regenerators; factors attributed in part to a rapid antioxidant response (18). These *in vitro* studies support the idea that intrinsic cellular mechanisms utilizing different signaling pathways, redox stabilizing reactions and possibly metabolic and epigenetic controls put *Acomys* fibroblasts in a state that primes them towards a regenerative response even in the absence of external factors such as tissue mechanics and inflammation. Since ROS generation and management is intimately linked to cellular metabolism, we hypothesized that ROS-reticent *Acomys* and *Oryctolagus* fibroblasts would possess a pro-glycolytic profile linked to mitochondrial physiology.

Here, we show that primary ear pinna fibroblasts from *Acomys* and *Oryctolagus* indeed show a pro-glycolytic metabolic state at baseline compared to *Mus* and *Rattus* fibroblasts, which instead exhibit a comparatively higher OxPHOS state typical of differentiated fibroblasts. The pro-glycolytic mitochondrial state is also marked by higher glucose sensitivity. We also find that *Acomys* fibroblasts possess mitochondria that are comparatively fewer in number, larger, rounded, mostly depolarized and tightly coupled. These results show that specific cellular metabolic states can be generated through variable mechanisms in evolutionarily distant mammals, supporting a cell state biased to favor regenerative healing and support the need to expand the range of mammalian models in biomedical research beyond the well-utilized few, especially in the field of wound healing.

## EXPERIMENTAL PROCEDURES

### Animals

Primary ear pinna cells (see below) were obtained from outbred laboratory mice (*Mus musculus* - ND4 Envigo) and spiny mice (*Acomys dimidiatus*) from our in-house breeding colonies at the University of Kentucky. While *Mus* were only fed 14% protein mouse chow (Tekland Global 2014, Harlan Laboratories, Indianapolis, IN), *Acomys* were fed the same mouse chow supplemented with black-oil sunflower seeds (Pennington Seed Inc., Madison, GA) in a 3:1 ratio. *Mus* and *Acomys* were also given access to water ad libitum and maintained in a 14-h light/10-hour dark cycle. Adult mice (4-month-old) and spiny mice (4-month-old) were sexually mature, and sex matched for all samples for each experiment. Sexually mature (16-week to 20-week-old) New Zealand rabbits (*Oryctolagus cuniculus)* and Sprague-Dawley rats (*Rattus norvegicus*) (16-week to 20-week-old) were obtained from the Division of Laboratory Animal Research, University of Kentucky. The University of Kentucky Institutional Animal Care and Use Committee (IACUC) approved all animal work described in this project under protocol 2019–3254.

### Tissue harvest, fibroblast isolation and *in vitro* culture

Primary fibroblasts were obtained from ear pinnae of all animals. Ear pinna tissue was harvested using 4 mm biopsy punches (Sklar Instruments, West Chester, PA) in the centers of the right and left ear pinnae. *Acomys* and *Mus* were anaesthetized with 3% vaporized isoflurane (v/v) (Henry Schein Animal Health, Dublin, OH) at 1 psi oxygen flow rate while Rattus and Oryctolagus were euthanized immediately prior to harvest. Tissues from each animal were pooled into separate 1.5 ml Eppendorf tubes. Single-cell suspensions were obtained via enzymatic (Trypsin-Dispase/Collagenase IV) and mechanical digestion (razor dicing/70 μm filtering) steps. The suspension was pelleted and the cell pellets re-suspended and cultured in complete DMEM (Corning^®^ 500 mL Dulbecco’s Modified Eagle’s Medium/Hams F-12 50/50 Mix) supplemented with 10% FBS (Hyclone), and 1% Antibiotic-Antimycotic (Gibco), at 37°C under 3% O_2_ and 5% CO_2_ conditions in 10 cm plates. Cells were cultured and passaged at ~80% confluency, with each subsequent passage seeded with 300,000 – 500,000 cells in a 10 cm plate. During passages, cells were counted using a hemocytometer. Fetal fibroblasts were isolated from embryos obtained from timed-pregnant females following euthanasia in accordance with approved animal care protocols. The ear buds and head skin were carefully dissected from the embryos and subjected to enzymatic digestion in 0.25% trypsin for 10 minutes at 37°C.Following digestion, the enzyme was neutralized using complete growth medium containing serum. Tissue was mechanically dissociated using a 20-gauge needle to obtain a single-cell suspension. Cells were then plated and cultured in Dulbecco’s Modified Eagle Medium/Ham’s F-12 (50/50 mix) supplemented with 10% fetal bovine serum (FBS, HyClone) and 1% Antibiotic-Antimycotic (Gibco). Cultures were maintained at 37°C, under hypoxic conditions (3% O_2_, 5% CO_2_) in 10 cm tissue culture dishes until reaching confluence. Additional detail on fibroblast isolations can be found here: dx.doi.org/10.17504/protocols.io.yxmvmb3kog3p/v1.

### MitoTracker Staining

Fibroblasts from all species were seeded into 24 well plates on cover slips at 5000 cells per well and cultured for 48 hours in complete DMEM (Corning^®^ 500 mL Dulbecco’s Modified Eagle’s Medium/Hams F-12 50/50 Mix) supplemented with 10% FBS (Hyclone), and 1% Antibiotic-Antimycotic (Gibco), at 37°C under 3% O2 and 5% CO2 conditions. Media was removed and the cells washed with HBSS, and then incubated with MitoTracker Red CMXRos (M7512, ThermoFisher scientific) staining media mix (0.3 μl staining stock/ 1 ml FBS-free DMEM) for 30 minutes. After incubation, cells were washed twice with PBS and then fixed for 15 minutes in the dark with warm 4% formaldehyde. The 4% formaldehyde was removed after fixation and the cells washed twice with PBS for 5 minutes. Chilled acetone was added to the cells at 500 μl/well and incubated for 5 minutes, after which it was removed, and the cells washed once with PBS. DAPI (1:2000/HBSS) was added to each well and incubated for 15 minutes. The DAPI solution was removed, and the cells finally washed with DI H2O. These coverslips were air dried and mounted on glass slides with Prolong Gold antifade reagent (Invitrogen) and imaged.

For Mitotracker green staining, fibroblasts were grown as above. After incubation, the media was removed, washed once with warm 1X HBSS. Before washing, Mitotracker green (M7514, ThermoFisher scientific) staining media mix was prepared by mixing 2 ul of the dye stock with 12 ml of FBS-free DMEM and warmed. The cells were incubated in the staining media mix for 30 minutes at the previously mentioned conditions. After incubation, the cells were washed once with 1X HBSS, HBSS added again, and the cells live imaged immediately (12 wells within 20 minutes) on an inverted incubator microscope (Olympus IX83). Additional detail for this protocol can be found here: dx.doi.org/10.17504/protocols.io.j8nlkyoo6g5r/v1

### Microscopy & Image Acquisition

Manual characterization of mitochondrial phenotypes was carried out using 40X fluorescent images taken using a IX83 microscope (Olympus, Tokyo, Japan) with a DP80 color camera on cellSens software (cellSens v1.12, Olympus Corporation). The same equipment was used in characterizing levels of senescence in cultured cells utilizing 20X fluorescent images with the DAPI, FITC and TEXAS RED channels for detecting nuclei, DNA strand breaks (γH2AX) and DNA replication (EdU) respectively. Cells were cultured on coverslips in 24 well plates and fixed before imaging. Cells positive for the individual markers were quantified using ~5 images captured at 20X magnification per sample, using an Olympus BX53 microscope with each image containing approximately 50 cells. With DAPI as a basal marker for all cells, marker-positive cells were quantified as a portion of all distinct DAPI positive cells. 40X images were used for characterizing mitochondrial phenotypes with ~8 images taken per sample (n=3/species) with >30 cells counted per sample. 20X images were taken for senescence detection with ~4 images taken per sample and >120 cells counted per sample (n=3/species).

### Transmission electron microscopy

Cells were fixed by replacing the growth medium with a fixative solution containing 4% paraformaldehyde and 3.5% EM-grade glutaraldehyde in 0.1 M Sorenson’s phosphate buffer at 4°C. After an initial fixation for 5 minutes at room temperature, the fixative was replaced with fresh solution and incubated at 4°C for 1 hour to ensure complete cross-linking. Following fixation, cells were thoroughly washed with 0.1 M Sorenson’s buffer and then post-fixed in 1% osmium tetroxide for 1 hour at 4°C. Samples were dehydrated in a graded ethanol series 50% through 100% for 5 minutes each, followed by two washes in absolute ethanol, all performed at 4°C. After dehydration, the cells were infiltrated with epoxy resin directly in the culture dish and transferred into beam capsules for overnight embedding. Polymerized blocks were sectioned at 80–90 nm thickness using a Reichert Ultracut E ultramicrotome. Sections were mounted on 300-mesh copper grids and stained with uranyl acetate and lead citrate to enhance contrast. Prepared grids were imaged at the University of Kentucky Electron Microscopy Center using a Talos F200X transmission electron microscope. Additional information for this protocol can be found here: dx.doi.org/10.17504/protocols.io.bp2l6zxxzgqe/v1.

### Fibroblast Oxygen Consumption and Extracellular Acidification Measurement

Measurements of cellular mitochondrial function, and glycolytic flux in fibroblasts were taken with the XF Cell Mito Stress Test (MST) Assay Kit (Agilent Technologies) and XF Glycolysis Stress Test (GST) Kit (Agilent Technologies) respectively, according to the manufacturer’s protocols (https://www.agilent.com/cs/library/usermanuals/public/XF_Cell_Mito_Stress_Test_Kit_User_Guide.pdf; https://www.agilent.com/cs/library/usermanuals/public/XF_Glycolysis_Stress_Test_Kit_User_Guide.pdf) and using the Seahorse Bioscience XFe96 Flux Analyzer (Agilent Technologies). With three biological replicates and three technical replicates for each cell line per species, cells were seeded at passage two into the 96-well Seahorse culture plate at 30,000 – 35,000 cells per well. Glycolytic stress tests, utilizing glucose, Oligomycin and 2-DG, and Mitochondrial stress tests utilizing ETC inhibitors (Oligomycin, FTTP, Rotenone and Antimycin A) were run on live cells 18–24 hours after seeding, per the protocol provided by the manufacturer. From the MST, OCR parameters such as proton leak, maximal respiration, basal respiration, and ATP-linked respiration were quantified, while the GST had ECAR parameters like glycolysis, glycolytic capacity, non-glycolytic acidification, and glycolytic reserve calculated.

### Mitochondrial membrane potential characterization: JC-1 Staining

P2 fibroblasts from *Acomys*, *Rattus*, *Mus* and *Oryctolagus* were plated on a 96-well black clear glass bottom plate and incubated for 24–48 hours. This dye is also cationic and labels mitochondria with respect to mitochondrial membrane potential reversibly. The JC-1 dye which is monomeric, specifically binds to all mitochondria and has a green fluorescence emission of ~529nm. Upon encountering “healthy” mitochondria with high membrane potential, the cationic dye accumulates in the mitochondrial matrix and forms aggregates, with a red fluorescence emission at ~590nm. The red/green fluorescence intensity ratios are then used to describe mitochondrial polarization state. The JC-1 dye is typically used as an indicator of mitochondrial health in response to different chemical stimuli, hinged on the principle that mitochondrial membrane potential disruption results in dysfunctional mitochondria, signifying early tell-tale signs of apoptosis. Here, this was used to compare mitochondrial polarization levels across the four species. For basal assessment of mitochondrial polarization, fibroblasts from the four species (4 biological replicates per species) were cultured on glass bottomed dark 24-well plates till they reached ~80% confluency, incubated with the dye for 30 minutes, washed and live-imaged. Fluorescence intensity in the red and green channels was quantified in the images taken using ImageJ.

To measure changes in mitochondrial membrane potential in response to mitochondrial inhibitors FCCP, and Oligomycin, cells were seeded on a 96 well black clear bottom plate. Cells were plated at a density of ~30,000 cells per well and incubated for 24 hours. During plating, allowance was made for controls and two treatment conditions. Cells were washed in 1X HBSS after which a staining media mix (2μM) was prepared and added at 100ul per well for 30 minutes. Halfway through the incubation period 1.5uM of Oligomycin and 10mM of FCCP were added to the treatment wells to complete the incubation. After a wash with warm 1X HBSS, the plates were scanned with excitation set at 485nm and emission detection set at 529nm for the green monomeric form and 590nm for the red aggregate form. Three washes with three corresponding readings were carried out. The ratio of green to red fluorescence signal intensities were used to quantify the levels of mitochondrial membrane potential. The values were normalized to ug of sample protein. Additional protocol detail can be found here: dx.doi.org/10.17504/protocols.io.rm7vz9xxrgx1/v1.

### Mitochondrial Isolation

Cultured fibroblasts were dislodged with trypsin (at P2), spun down (1500 rpm for 5 minutes) five minutes after the reaction was stopped with FBS-containing DMEM. The supernatant was poured off and pellets kept on ice. 1 ml of Mitochondria Isolation Buffer (MIB) was added to the pellet and the pellet re-suspended. The suspension was spun at 400 rcf for 5 minutes, after which the supernatant was taken out and 450 μl of EDTA added to the pellet and re-suspended. Samples were broken up using a Nitrogen cell disruptor (1250psi for 10 minutes). The samples cell lysates were passed twice through a 26-gauge syringe, topped up with 1.5 ml of Mitochondria Isolation Buffer, spun at 1300×g for 3 minutes at 4°C and the supernatant transferred into new tubes. These were then spun at 4°C at 1300 × g for 10 minutes. A Ficoll gradient was made up using 25 ml of STE (8.55g of sucrose, 32 ul of 0.5 M EDTA, 80 ul of 1 M Tris and topped up to 25 ml with dIH_2_O) and a Ficoll stock (10 ml STE and 17.5 ml 20% Ficoll). The mitochondria were added to and then spun down in the Ficoll gradient (2 ml 7.5%/ 2 ml 10%). After spinning at 32 000 rpm for 30 minutes, the Ficoll supernatant was discarded, and the pellet resuspended in 1 ml MIB and spun at 13,000 rcf for 10 minutes. The supernatant was then discarded and the pellets (purified mitochondria) resuspended in MIB and quantified using a BSA assay. Additional protocol detail can be found here: dx.doi.org/10.17504/protocols.io.81wgbwxr1gpk/v1.

### Electron Transport Chain Protocol (ETCP)

A modified version of the Respirometry in frozen samples (RIFS) assay (32) called the Electron transport chain protocol (ETCP) (33) was carried out using n=4 biological replicates/species (aka four independently derived primary lines per species): i.e., frozen mitochondria previously isolated from cultured *Acomys*, *Mus*, *Rattus* and *Oryctolagus* ear pinna fibroblasts (P2). After thawing the samples, 25ul of Isolation buffer was added to all samples and gently resuspended. 4ul of mitochondrial mix was loaded per well (2 technical replicates) and the protein concentration for each sample was measured using the BCA assay. Seahorse XF 96 well cartridge ports were prepared as follows: Port A (ETC 1): 3220ul Mitochondrial Respiration buffer w/o BSA, 2.8ul Rotenone, 280ul succinate; Port B: 3500ul Mitochondrial Respiration buffer w/o BSA, 3.5ul Antimycin A; Port C: 3325ul Mitochondrial Respiration buffer w/o BSA, 88ul Ascorbate, 88ul TMPD; Port D: 3033ul Mitochondrial Respiration buffer w/o BSA, 480ul Azide. Each well had each port loaded with 25ul of the port mixes. With the Seahorse XF 96 cell culture plate on ice, 75ul mitochondrial samples (2ug protein) were loaded per well, after which the plate was balanced and spun at 3214 rcf for 10 minutes at 4°C. 11ml of the Alamethicin Assay solution (10.6ml Mitochondrial Respiration buffer w/o BSA, 40mg/ml Alamethicin 9.63ul, 1.5mM NADH 385ul, 10 uM cytochrome c 48ul) was prepared with 100ul of the assay solution added to each well and ran on the Seahorse XF 96 Analyzer immediately. With the contents of Ports A-D injected sequentially over a period of 60 minutes, Complex 1, 2 and 4 activities were determined. Additional protocol detail can be found here: dx.doi.org/10.17504/protocols.io.6qpvrw39olmk/v1.

### Statistical analyses

The in vitro experiments described had at least 3 biologically independent samples (cell lines from separate animals) and a minimum of 3 technical replications per said sample utilized measured; we did not calculate power. Data were initially compiled using Microsoft Excel with JMP (version Pro 17.1.0, SAS Institute Inc., https://www.jmp.com/en/software/data-analysis-software, RRID:SCR_014242) used to perform all statistical analyses. Data from Seahorse assay, respirometry, fluorescence signal intensity experiments were presented as mean ± SD and had their statistical significance determined using one-tailed or two-tailed ANOVA with all post hoc pairwise comparisons made with the students *t*-test. Alpha was set at 0.05 to assess significance for all statistical analyses.

## RESULTS

### Fibroblasts from regenerating mammals exhibit a relatively low basal metabolic rate and high glycolytic rate

Based on previous data demonstrating strong resistance to oxidative stress in *Acomys* and *Oryctolagus* ear pinna fibroblasts (18), we hypothesized this result could reflect a reduced metabolic state not typically observed in mouse and human fibroblasts. We ran conventional Seahorse Mitochondria Stress and Glycolytic Stress tests (MST and GST) using primary ear pinna fibroblasts from highly regenerative (*Acomys*, *Oryctolagus*) and non-regenerative (*Rattus*, *Mus*) mammals cultured for two passages (see Methods).

**Figure 1.**
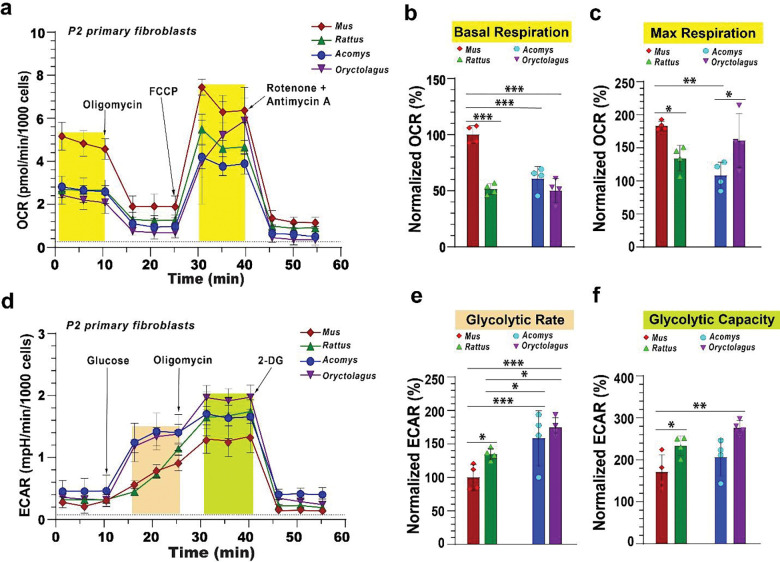
Fibroblasts from regenerating mammals exhibit a high glycolytic rate. MS-mitochondrial stress tests **(a-c)** and GST-Glycolytic tests **(d-f)** run on Seahorse XFe96 analyzer using primary ear pinna fibroblasts from *Acomys*, *Oryctolagus*, *Mus* and *Rattus*. Oxygen consumption rates (OCR) and extracellular acidification rates (ECAR) were normalized to the basal respiration and glycolytic rates in *Mus*. Basal respiration levels were significantly higher in *Mus* compared to the other three species (ANOVA, *F*=28.1749, *p*<0.0001) (a-b). Maximal respiration was significantly different among species (ANOVA, *F*=6.775, *p*=0.0063), with higher rates in *Mus* compared to *Acomys* and *Rattus* but not *Oryctolagus* (*p* = 0.2286) (c). Maximal respiration in *Oryctolagus* was significantly higher compared to *Acomys*. Fibroblasts from *Acomys* and *Oryctolagus* exhibited significantly higher glycolytic rates compared to the non-regenerating species (ANOVA, *F*=23.3301, *p*< 0.0001) (d-e). Also, *Acomys* and *Oryctolagus* fibroblasts exhibited significantly higher glucose sensitivity compared to *Mus* and *Rattus*, responding rapidly to glucose injection 10 to 15 minutes faster (d). Individual data points represent biological replicates for each species (n=4). FCCP: carbonyl cyanide 4-(trifluoromethoxy)phenylhydrazone. 2-DG: 2-Deoxy-d-glucose. (* *p*<0.05, ** *p*<0.001, *** *p*<0.0001).

Using sequential injections of oligomycin, FCCP, and rotenone/antimycin A to inhibit ATP synthase, disrupt the mitochondrial proton gradient, and inhibit Complex V, I/III activity respectively, we measured oxygen consumption rates and calculated basal respiration, maximal respiration, and specific respiratory capacity (Fig. 1a–c, Supp. Fig 1). *Mus* fibroblasts exhibited significantly higher oxygen consumption at baseline compared to *Acomys*, *Rattus* and *Oryctolagus* fibroblasts (Fig 1a–b). Despite having a low basal metabolic rate, in response to FCCP *Oryctolagus* fibroblasts rapidly increased oxygen consumption exhibiting maximal respiration levels matching that observed in *Mus* and *Rattus* (Fig. 1a–c). In contrast, maximal respiration and specific respiratory capacity (maximal respiration – basal respiration) were significantly lower in *Acomys* compared to the other species (Fig. 1a–c and Suppl. Fig. S1).

In parallel with the MST, we ran a glycolytic stress test measuring extracellular acidification rates (ECAR) while sequentially injecting glucose, oligomycin and 2-deoxyglucose to calculate glycolytic rate, glycolytic capacity, glycolytic reserve and non-glycolytic acidification (Fig. 1d). Measuring the amount of extracellular acid produced by cells from all four species we observed significantly higher glycolytic rates in cells from the regenerating species (Fig. 1d–e). Although we did not detect significant differences among other GST parameters between regenerators and non-regenerators, we observed that *Acomys* and *Oryctolagus* showed a similarly high sensitivity to glucose, responding immediately to extracellular glucose while *Mus* and *Rattus* exhibited a delayed reaction (Fig 1d). Taken together, these results show that *Acomys* and *Oryctolagus* fibroblasts exhibit a low metabolic rate and high glycolytic rate, while *Mus* and *Rattus* fibroblasts exhibit high maximal respiration and low glucose sensitivity. *Oryctolagus* fibroblasts exhibit an increased sensitivity to glucose but also an ability to ramp up mitochondrial respiration abruptly in response to an acute energy deficit.

### Functional mitochondria in Acomys fibroblasts resemble megamitchondria

**Figure 2.**
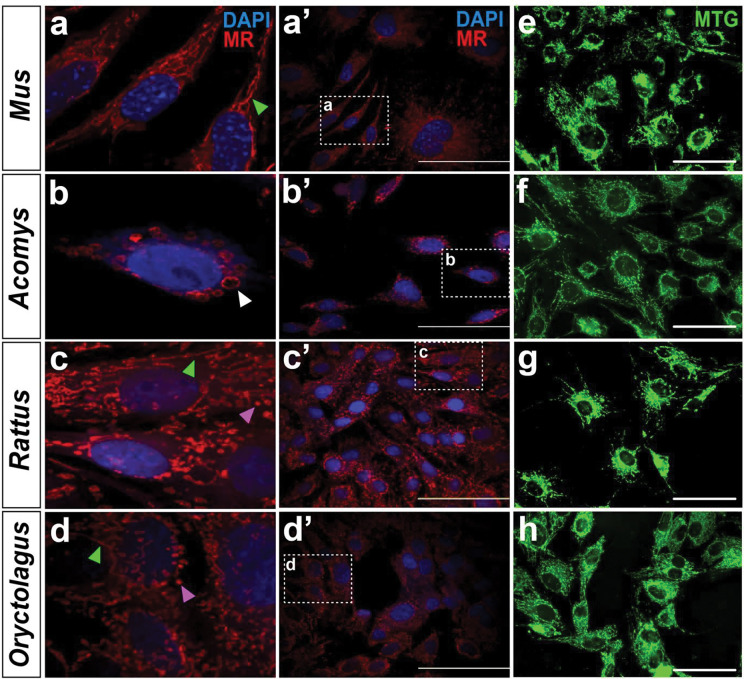
Active mitochondria in healthy *Acomys* cells resemble megamitochondria. **(a’-d’)** Representative images of fixed fibroblasts from *Mus, Acomys, Rattus* and *Oryctolagus* stained with Mitotracker CMXROS red dye (MR). (a-d) Inset images at 60x magnification show classic tubular mitochondria in *Mus*, *Rattus* and *Oryctolagus* (green arrows), whereas active mitochondria in *Acomys* resemble megamitochondria with a large, spherical phenotype (white arrows) (Scale bars: 80 μm). Small, round mitochondria were also visible in *Rattus* and *Oryctolagus* (purple arrows). (e-h) Representative live images of cultured P2 ear pinna fibroblasts from *Mus, Acomys, Rattus* and *Oryctolagus* stained with Mitotracker green dye (MTG) showing all mitochondria (Scale bars: 20 μm).

Next, we asked if mitochondria in regenerating species exhibited morphological differences that could explain their observed predilection for glycolysis using CMX-Ros Mitotracker red (MR) and Mitotracker green (MG). MR labels mitochondria with a high resting membrane potential which are often interpreted as active. *Mus* mitochondria stained with MR exhibited the classical morphology typified by tubular networks extending throughout the cytoplasm (Fig. 2a–a’). In stark contrast, *Acomys* fibroblasts presented large, spheroid mitochondria clustered around the nucleus which were relatively few in number (~20/cell) and resembled megamitochondria (34) (Fig. 2b–b’). *Oryctolagus* and *Rattus* fibroblasts exhibited tubular mitochondrial networks more in line with *Mus*, although spherical mitochondria were also observed randomly dispersed in the cytoplasm (Fig. 2c–d’). The circular mitochondria observed in *Oryctolagus* and *Rattus*, however, were small, approximately the size of fragmented mitochondrial tubes in contrast to the megamitochondria found in *Acomys* (35) (Fig. 2b–d’).

To determine if the megamitochondria phenotype observed in *Acomys* was strictly associated with active mitochondria, we used MG to label mitochondrial protein cysteine residues that accumulate in the mitochondrial matrices independent of membrane potential to label all mitochondria (36). While MG staining revealed classic tubular networks in *Acomys*, it did not stain megamitochondria (Fig. 2f). In *Mus*, *Rattus* and *Oryctolagus*, the mitochondrial networks observed with MG mirrored that observed with MR (Fig. 2e, g–h). Considered together, these results demonstrate that while *Mus*, *Rattus* and *Oryctolagus* possess extensive networks of mostly active, tubular mitochondria, these same networks in *Acomys* fibroblasts are likely dysfunctional, leaving the megamitochondria to generate ATP. This unique spherical morphology tied to active mitochondria in *Acomys* may help explain the absence of a dramatic increase in oxygen consumption in response to a sudden energy demand mimicked by FCCP injection, in that such spherical mitochondria have been linked to poor respiratory measures due to underdeveloped cristae formation (37).

### Acomys mitochondria are inherently depolarized but highly efficient at producing ATP

**Figure 3.**
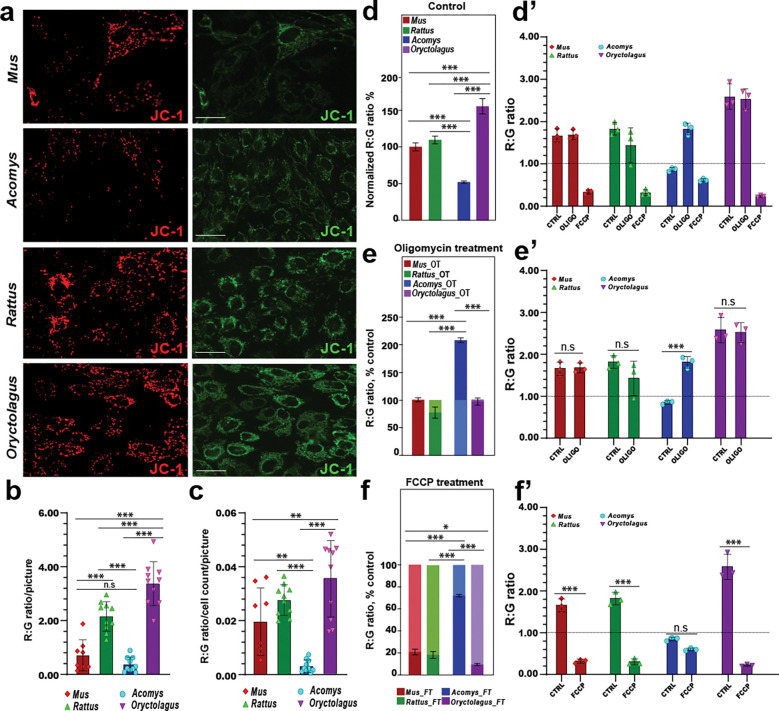
*Acomys* mitochondria have exhibit low membrane potential and are tightly coupled. **(a)** Live fluorescence images of P2 ear pinna fibroblasts from *Mus*, *Acomys*, *Rattus* and *Oryctolagus* stained with JC-1 and imaged in red and green channels at 20x magnification, (Scale bars = 20 μm). **(b-c)** Red:green fluorescence intensity ratios measured from images **(a)** and normalized against total cell count were used to estimate mitochondrial membrane potential. **(d-f’)** Due to the photolability of the JC-1 dye, fluorescence intensity was better measured using a plate reader to capture acute changes in membrane potential responding to mitochondrial inhibitors FCCP and Oligomycin. Red:green signal ratios were quantified and normalized to total μg protein and pegged against untreated *Mus* (**d-f**). *Acomys* fibroblasts exhibited the lowest membrane potential among all four species (51.9% of *Mus*), with *Oryctolagus* having the highest (155.4% of *Mus*), with *Rattus* (109%) not significantly different from *Mus* (ANOVA, *F*= 167.4372, *p*<0.0001) (**d-d’**). Membrane potential in *Acomys* mitochondria increased two-fold in response to oligomycin, while in *Rattus*, *Mus*, and *Oryctolagus* exhibited no significant change compared to basal membrane potential (ANOVA, *F*= 10.0687, *p*=0.0006) (**e, e’**). FCCP treatment caused an insignificant decrease in mitochondrial membrane potential in *Acomys*, compared to *Mus*, *Oryctolagus* and *Rattus* (*p*_Mus_<0.001, *p*_Oryctolgagus_<0.001, *p*_Rattus_<0.001,) which dropped significantly to 20.4%, 9.2%, and 17.8% respectively of their basal red:green ratios in response to the uncoupler (ANOVA, *F*= 60.1244, *p*<0.0001) (**f, f’**). Individual data points represent biological replicates per species (n = 3). (* *p*<0.05, ** *p*<0.001, *** *p*<0.0001).

Our morphological investigation suggested membrane polarization as a source of variation across species that might explain the unique metabolic state observed in spiny mouse cells. Because Mitotracker red and green stains cannot be visualized simultaneously, we turned to JC-1 to better quantify mitochondrial mass and mitochondrial functionality. Using microscopy to visualize JC-1 green fluorescence, images from all four species mirrored MG staining (Fig. 3a). However, while fluorescence in the red channel for *Mus*, *Rattus*, and *Oryctolagus* showed strong synchrony with that of fluorescence in the green channel, mitochondria in *Acomys* exhibited a much weaker signal across the entire field of view (Fig. 3a). Using GFP (529 nm) and RFP (590 nm) fluorescence, we quantified red to green fluorescence intensity ratios and found that *Acomys* fibroblasts had the lowest red/green fluorescence intensity ratio whether we did or did not normalize for cell number (Fig. 3a–c). Still, given the photolability of the JC-1 dye which could in principle affect our ratio calculations, we opted to also capture fluorescence using a plate reader.

Culturing cells to ~80% confluency, we incubated with JC-1for 30 minutes, washed and then simultaneously detected emissions at 529 nm and 590 nm (Fig. 3d–f’). Using this method, membrane potential (red to green signal ratio) in *Acomys* fibroblasts was significantly lower compared to the other three species (Fig. 3d–d’). We also exposed biological replicates to two additional treatments: oligomycin and FCCP creating hyperpolarized and depolarized mitochondria respectively (Fig. 3e–f’). In response to oligomycin, *Acomys* mitochondria exhibited a two-fold increase in membrane potential as expected since inhibiting ATP synthase produces a net positive build-up of protons in the mitochondrial intermembrane space resulting in mitochondrial hyperpolarization (Fig. 3e–e’). Interestingly, *Rattus*, *Mus*, and *Oryctolagus* fibroblasts showed no significant change in mitochondrial membrane potential in response to oligomycin treatment suggesting mitochondria, on average, were hyperpolarized in culture (Fig. 3e–e’). The inverse was seen for the FCCP treatment, where proton gradient disruption triggered dramatic depolarization in *Mus*, *Rattus* and *Oryctolagus* as expected, however *Acomys* fibroblasts showed no significant decrease in membrane potential (Fig. 3f–f’). Thus, our JC-1 data further support that *Acomys* mitochondria are generally low functioning, and yet they are very sensitive to ATP synthase inhibition, indicating they are highly efficient with respect to ATP production.

### Frozen Isolated mitochondria from Acomys and Oryctolagus share low Complex I, II, and IV dependent respiration

**Figure 4.**
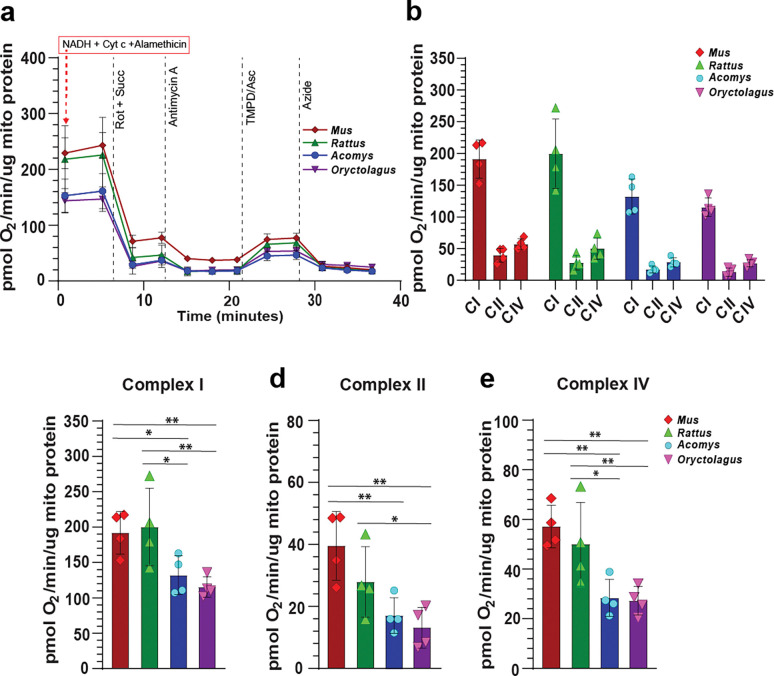
*Mus* and *Rattus* fibroblasts have significantly elevated ETC complex activity compared to *Acomys* and *Oryctolagus*. **(a-e)** Representative seahorse traces of oxygen consumption per microgram of mitochondrial protein quantified in isolated, frozen mitochondria from P2 ear pinna fibroblasts using ETCP, a modified version of the RIFS respirometry assay. Frozen mitochondria were assayed utilizing alamethicin, NADH and supplementing lost cytochrome c. *Oryctolagus* and *Acomys* mitochondria had lower (CI) Complex I-dependent respiration compared to *Mus* and *Rattus* (ANOVA, *F*=5.8468, *p*=0.0106). Similarly, (CII) Complex II-dependent respiration in *Oryctolagus* was again lower than in the non-regenerators. *Mus* Complex II respiration was higher than in *Acomys*, although the difference between *Acomys* and *Rattus* was not significant (ANOVA, *F*=6.8119, *p*=0.0062). Cytochrome c oxidase (CIV) respiration was significantly higher in the non-regenerators than the two regenerators (ANOVA, *F*=8.3265, *p*=0.0029). For all three Complex-dependent respiration measurements, individual data points represent biological replicates/species, (n = 4). An average of four technical replicates were used to represent each biological replicate. Post-hoc pairwise comparisons were carried out using the students’ *t*-test. (* *p*<0.05, ** *p*<0.001, *** *p*<0.0001).

After observing key differences in mitochondrial membrane potential across all four species, we set out to quantify oxygen consumption from isolated mitochondria. Isolated mitochondria were frozen and assayed using the electron transport chain protocol (ETCP) (33), where we utilized alamethicin and supplemented lost cytochrome c. This method allows assessment of mitochondrial complex function without a contribution from the proton gradient (32), which is disrupted after successive freeze-thawing cycles (38). Despite losing the proton-motive force, activity from complexes I, II and IV are observed, allowing the assessment of ETC complex activity in archived samples, years after collection (32, 33). Performing the ETCP assay we observed *Oryctolagus* mitochondria to possess significantly reduced complex I, II and IV activities (oxygen consumption per microgram of mitochondrial protein) compared to *Mus* and *Rattus* (Fig. 4a–e). Similarly, *Acomys* activity in complexes I, II and IV was significantly lower compared to the non-regenerators (Fig. 4a–e). When complex activity is considered in toto across the entire treatment time course, a stark dichotomy emerges separating the regenerators and non-regenerators; *Acomys* and *Oryctolagus* mitochondria show consistently lower oxygen consumption per ug of mitochondrial protein even in response to TMPD and Ascorbic acid, which restored oxygen consumption to levels observed prior to Antimycin A injection (Fig. 4a). Taken together, in the absence of a protonmotive gradient, mitochondria from regenerators exhibit intrinsically lower oxygen consumption compared to non-regenerators.

### Megamitochondria persist across lifespan and remain resilient to oxidative stress in spiny mice fibroblasts

**Figure 5.**
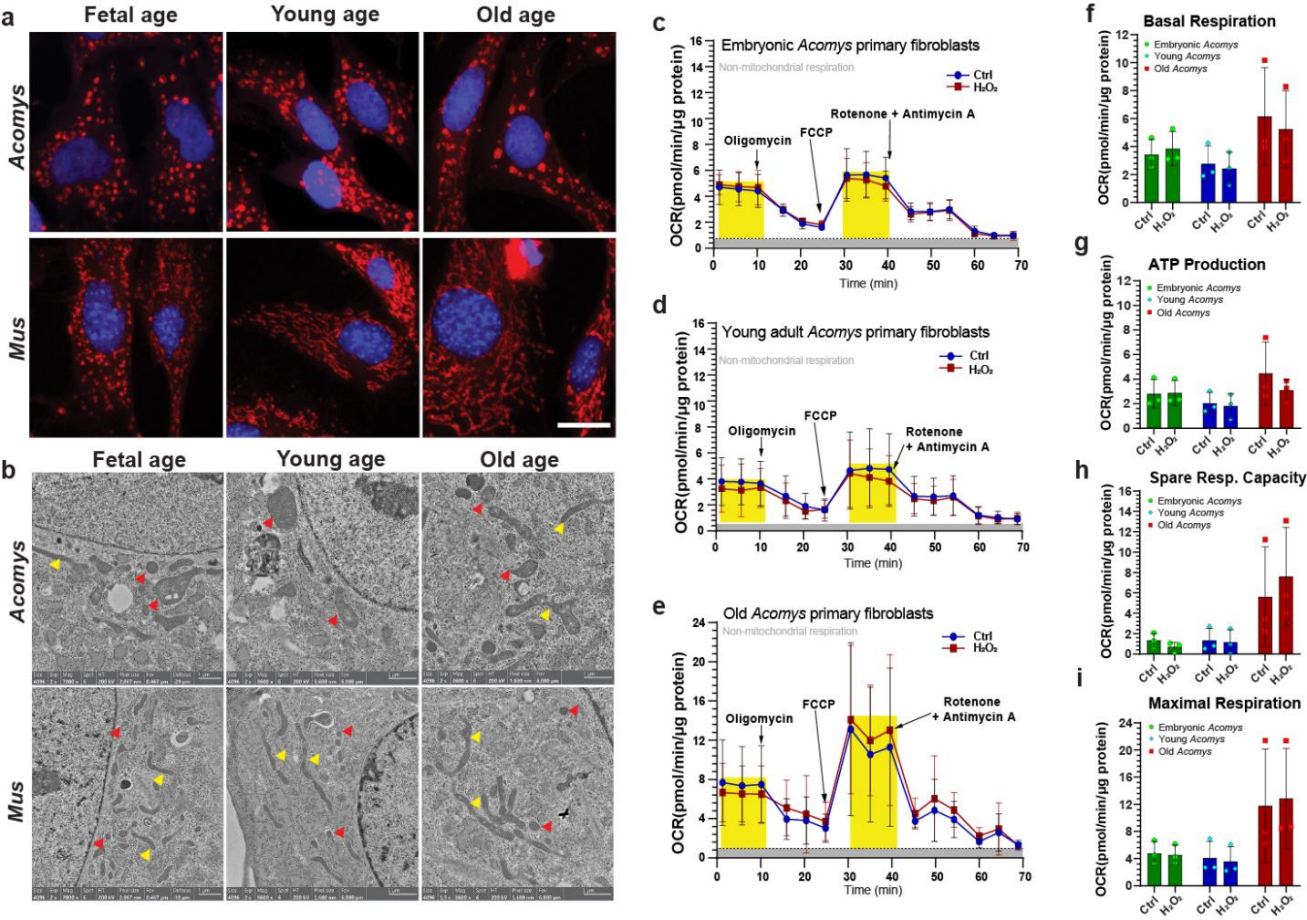
*Acomys* cells are highly resistant to oxidative stress regardless of age and megamitochondria are persistent across lifespan. (a) Primary ear pinna fibroblasts from fetal, young and old *Mus* and *Acomys* stained with MitoTracker Red (scale bar = 20 μM). *Acomys* fibroblasts present megamitochondria across all ages. Small, circular mitochondria are also present in *Mus* embryonic fibroblasts, but mitochondria adopt a tubular morphology after birth (a). Electron micrograph images showing megamitochondria in *Acomys* and tubular mitochondria in *Mus* (red arrows = round mitochondria, yellow arrows = tubular mitochondria). Mitochondrial function in *Acomys* is unaffected by 300 μM H_2_O_2_ treatment across different age classes as measured by OCR (oxygen consumption rates) via the Seahorse mitochondrial stress test. There aren’t significant changes in Basal respiration (ANOVA, *F*=0.1576, *p* = 0.8559), ATP production (ANOVA, *F*= 0.4455, *p*= 0.6507), Maximal respiration (ANOVA, *F*= 0.0449, *p* = 0.9563), and Spare respiration capacity (ANOVA, *F*= 0.3455, *p*= 0.7147), in response to H_2_O_2_ treatment. Individual data points represent biological replicates (n = 3) per age class/species. Post-hoc pairwise comparisons were carried out using the students’ *t*-test. FCCP: Carbonyl cyanide-p-trifluoromethoxy phenyl hydrazone.

To further assess how cellular metabolic state and mitochondrial morphology might functionally affect fibroblast phenotype we examined *Mus* and *Acomys* cells isolated from embryonic (*Mus*- E12.5, *Acomys*- E20–25), young (*Mus*-3 months, *Acomys*- 6 months) and old (*Mus*- 2+ years, *Acomys*- 4+ years) animals (Fig. 5a–f). Staining *Mus* cells with Mitotracker red, we observed a network of small, round and tubular mitochondria in E12.5 MEFs that transitioned into the largely tubular network associated with cells from adult animals (young or old) (Fig. 5a). In stark contrast, MR staining of *Acomys* fibroblasts across lifespan revealed that megamitochondria were the predominant, active form around the nucleus and in the cytoplasm independent of age (Fig. 5a). Subsequent cellular TEM analysis across all ages reinforced the retention of megamitochondria in *Acomys* fibroblasts (Fig. 5b). These data support that the megamitochondria phenotype found in healthy *Acomys* fibroblasts is independent of development and aging supporting evolved functionality related to cellular physiology.

Previous work in our lab uncovered that fibroblasts from young spiny mice were resilient to very high levels of hydrogen peroxide (18) and thus we exposed cells from all ages to 300μm H_2_O_2_ and assessed mitochondrial stress (Fig. 5c–i). Two trends were noted. First, cells from the oldest animals exhibited a slight, but insignificant increase in basal respiration (Fig. 5f), spare respiratory capacity and maximal respiration (Fig. 5h–i). Despite these trends, when cells from all age groups were exposed to 300μm H_2_O_2_ there was no significant alteration in metabolic function underscoring the resiliency of spiny mouse cells to oxidative stress (Fig. 5c–i). Together, our data supports that megamitochondria phenotype, low metabolic rate, and high resilience to oxidative stress are features of spiny mouse cells that are invariant across lifespan. The degree to which these cellular phenotypes functionally support regeneration awaits further testing.

## DISCUSSION

This study set out to metabolically characterize the phenotype of primary fibroblasts from two highly regenerative mammals (*Acomys* - spiny mice and *Oryctolagus* - rabbits) compared to fibroblasts from non-regenerative *Mus* and *Rattus*. By quantifying cellular oxygen consumption (OCR) and extracellular acidification (ECAR) in primary ear pinna fibroblasts we found that *Acomys* and *Oryctolagus* cells preferentially used glycolysis to support basal metabolism in culture and exhibited increased sensitivity to glucose compared to poorly regenerative *Mus* and *Rattus*. Conversely, *Mus* fibroblasts showed high basal respiration, maximal respiration, and spare respiratory capacity. We also discovered that mitochondria in *Acomys* fibroblasts lacked the tubular, networked morphology typically seen in fibroblasts from adult mammals, including humans. Specifically, *Acomys* mitochondria resembled megamitochondria (34, 35), presenting as large, active spheroids, whereas mitochondria in *Mus* fibroblasts were almost completely tubular, exhibiting extensive networks dispersed throughout the cytoplasm. *Rattus* and *Oryctolagus* were similar to *Mus* but also exhibited some small spherical mitochondria.

Low respiring, small, circular mitochondria are usually associated with embryonic stem cells (24) where they are ascribed poor respiratory measures due to underdeveloped cristae formation and an anoxic environment (25). Similarly, megamitochondria exhibit a spherical morphology and glycolytically biased metabolic state, although this phenotype has been exclusively associated with disease states or hypoxia (34, 35). To explore how form might echo function in primary fibroblasts, we employed a JC-1 assay to measure intracellular mitochondrial membrane potential. To understand how the JC-1 assay helps us quantify electron transport chain (ETC) function, consider the following analogy. Utilizing FCCP (membrane depolarizer) and oligomycin (proton channel blocker) allows us to conceptualize the entire ETC as a tap-connected garden hose with an operable spout. Turning on the tap represents complex I & II reactions, with electron transfer from NADH (and FADH_2_) to ubiquinone simultaneously pumping protons from the mitochondrial matrix into the intermembrane space. The differential membrane potential drives protons back into the matrix via ATP synthase, which couples ATP production to proton pumping back into the matrix as water flows through the hose. Administering oligomycin stops up the garden hose at the outflow thus generating a significant increase in mitochondrial membrane potential due to the buildup of protons in the intermembrane space compared to the matrix. Adding FCCP is analogous to punching holes in our hose which decreases net pressure of water flowing through the hose causing membrane potential to fall. After oligomycin treatment, a spike in membrane potential was only observed in *Acomys*, suggesting that the mitochondria in the other species were maximally polarized most likely due to a higher respiratory rate. When FCCP was added, we observed a significant drop in mitochondrial membrane potential in *Mus*, *Rattus* and *Oryctolagus*. Surprisingly, adding FCCP did not decrease membrane potential in *Acomys* suggesting that *Acomys* mitochondria are intrinsically depolarized. Together, these data support that *Acomys* megamitochondria are in fact tightly coupled and highly efficient resembling a metabolic state sometimes observed in ESCs (39).

To further dissect function and efficiency of the ETC in these cells, we utilized a ETCP assay to measure intrinsic mitochondrial oxygen consumption and ATP production of individual complexes in the absence of a proton gradient (32, 33). Deploying this assay, we observed a trend that split mitochondria from the four species into two groups: regenerating and non-regenerating exhibiting low and high oxygen consumption respectively. *Mus* and *Rattus* showed consistently higher levels of oxygen consuming capacity with high activity observed for complexes I, II and IV. Importantly, while our JC-1 data demonstrated that *Oryctolagus* mitochondria appeared similar to *Mus* and *Rattus* with respect to membrane potential, data from our ETCP analysis provides a potential explanation for this discrepancy. Here we observed decoupling between high membrane potential observed in rabbit fibroblasts and actual oxygen consumption capacity per microgram of mitochondrial protein. Although mitochondria from rabbit ear pinna fibroblasts exhibited high membrane potential, this does not reflect in their low basal respiration levels and high glycolytic flux mirroring that of *Acomys*. The high mitochondrial membrane potential observed in *Oryctolagus* interestingly supports their high spare respiratory capacity, which *Acomys* do not share. Furthermore, our data suggests that low basal respiration and maximal respiration levels (forced by FCCP) observed in *Acomys* are not just the result of low mitochondrial numbers, but instead, these mitochondria are depolarized and intrinsically inefficient (per ug of mitochondrial protein) at consuming oxygen explaining their low oxidative phosphorylation levels. Further studies will be necessary to determine a deeper mechanistic explanation for the phenotypes observed in *Oryctolagus* i.e., the discrepancy between high glycolytic rates, the low basal respiration levels, and intrinsic inefficiency in oxygen consumption despite having higher mitochondrial membrane potential.

Considering the influence of aging on metabolic phenotype, mitochondrial labelling and TEM imaging carried out indicated that *Acomys* fibroblasts maintain the megamitochondria-like phenotype across lifespan. With previous studies showing that this phenotype is typically associated with diseases (34), artificial induction by hypoxia (35) and exposure to free radical-generating compounds (40), this mitochondrial phenotype is not a positive sign of cellular health. Here we report that *Acomys* primary fibroblasts possessing megamitochondria, were far from unhealthy and actively resist senescence inducing stressors. A key finding here is that fibroblasts from fetal and old *Acomys* concurrently resisted ROS-induced mitochondrial dysfunction as earlier observed young adult fibroblasts (18). Therefore, this concurrent persistence of cellular resilience and metabolic phenotype gives more credence to the hypothesis that this metabolic signature is a strong causal factor. Also, it could be argued that this mitochondrial phenotype in *Acomys* is induced by hypoxic culture conditions (3% O_2_). It is key to note that this oxygen concentration better represents physiological levels in vivo, and that cells from the other species observed were cultured together in the same conditions but did not share this mitochondrial phenotype and that this mitochondrial state in Acomys may be a species-specific adaptation.

Research performed over the last decade, primarily in laboratory mice, identified that certain pre-injury fibroblast phenotypes appeared to support regenerative healing over fibrotic repair (41–45). While some of these studies connected phenotype to specific signaling pathways (e.g., Wnt-signaling activation during regeneration) (41, 46), others uncovered differential mechano-sensing (47, 48) or inflammatory priming (49) as key drivers of skin regeneration. Although deploying these approaches has yet to successfully alter healing of more complex tissue injuries, such as large (>4mm) ear pinna defects or amputated P2 digit tips, the general principle that specific fibroblast phenotypes are at least partially required for complex tissue regeneration is well-supported from contemporary regeneration studies (30, 50, 51). Identifying molecular regulators of fibroblast phenotype beyond transcriptomic profiling remains poorly characterized, but specific metabolic characteristics are emerging as key drivers underlying cellular phenotypes. Although it can be argued that this pro-glycolytic state observed in *Acomys* and *Oryctolagus* may largely be responsible for a potential for increased cell proliferation in the wound healing context, a previous study indicated that non-regenerative, OxPhos-dependent *Rattus* fibroblasts, although susceptible to ROS-induced senescence, were highly proliferative even more than observed in *Acomys* and the other non-regenerator, *Mus* (18), suggesting that this metabolic phenotype may be contributing mainly towards cellular resilience, which here we see, is maintained into old age, concurrently with regenerative ability (Aloysius et al., in prep), tying the cellular resilience to regenerative outcomes. Further studies can be carried out in rabbits to observe if their regenerative and fibroblast metabolic phenotypes are conserved with aging as seen in *Acomys*.

The main findings of this study point to the need for expanding the use of non-traditional mammalian models, especially for understanding how tissue repair, inflammation and aging are connected at the molecular level. Moreover, the close phylogenetic proximity of these species has created a paradigm to uncover key genomic, molecular and cellular features that can explain the emergence of regeneration in mammals. Here, two phylogenetically distant mammals share the trait of regenerative ability, coinciding with a shared rapid response to oxidative stress, and finally a shared glycolytic state, which is in turn acquired via different mechanisms involving mitochondrial function and intrinsic mitochondrial ROS production. Further tests screening fibroblasts from multiple mammalian models across different ecosystems and phylogenies would help in creating a mosaic of cellular traits which could support regenerative ability, consisting of different species-specific molecular mechanisms, with proliferative, ROS-adaptive, highly glycolytic, metabolically plastic, low collagen I-producing, non-fibrotic tissue fibroblasts as the pivotal common denominator.

## Supplementary Material

Supplement 1

## Data Availability

The data, code, protocols, and key lab materials used and generated in this study are listed in a Key Resource Table alongside their persistent identifiers (Supplementary Tables). No code was generated for this study; all data cleaning, preprocessing, analysis, and visualization were performed using Microsoft Excel and JMP.
