# Supplementary material for: Active mitochondria in healthy spiny mouse fibroblasts resemble megamitochondria and remain resilient across lifespan
Source: bioRxiv. 2025 Oct 4:2025.10.02.680123. Preprint. [Version 1] doi: 10.1101/2025.10.02.680123 (PMC12621865; doi:10.1101/2025.10.02.680123)
Supplement: Supplement 1 [file NIHPP2025.10.02.680123v1-supplement-1.pdf]

bioRxiv preprint doi: https://doi.org/10.1101/2025.10.02.680123; this version posted October 4, 2025. The copyright holder for this preprint (which was not certified by peer review) is the author/funder, who has granted bioRxiv a license to display the preprint in perpetuity. It is made available under aCC-BY 4.0 International license.

Supplementary figures

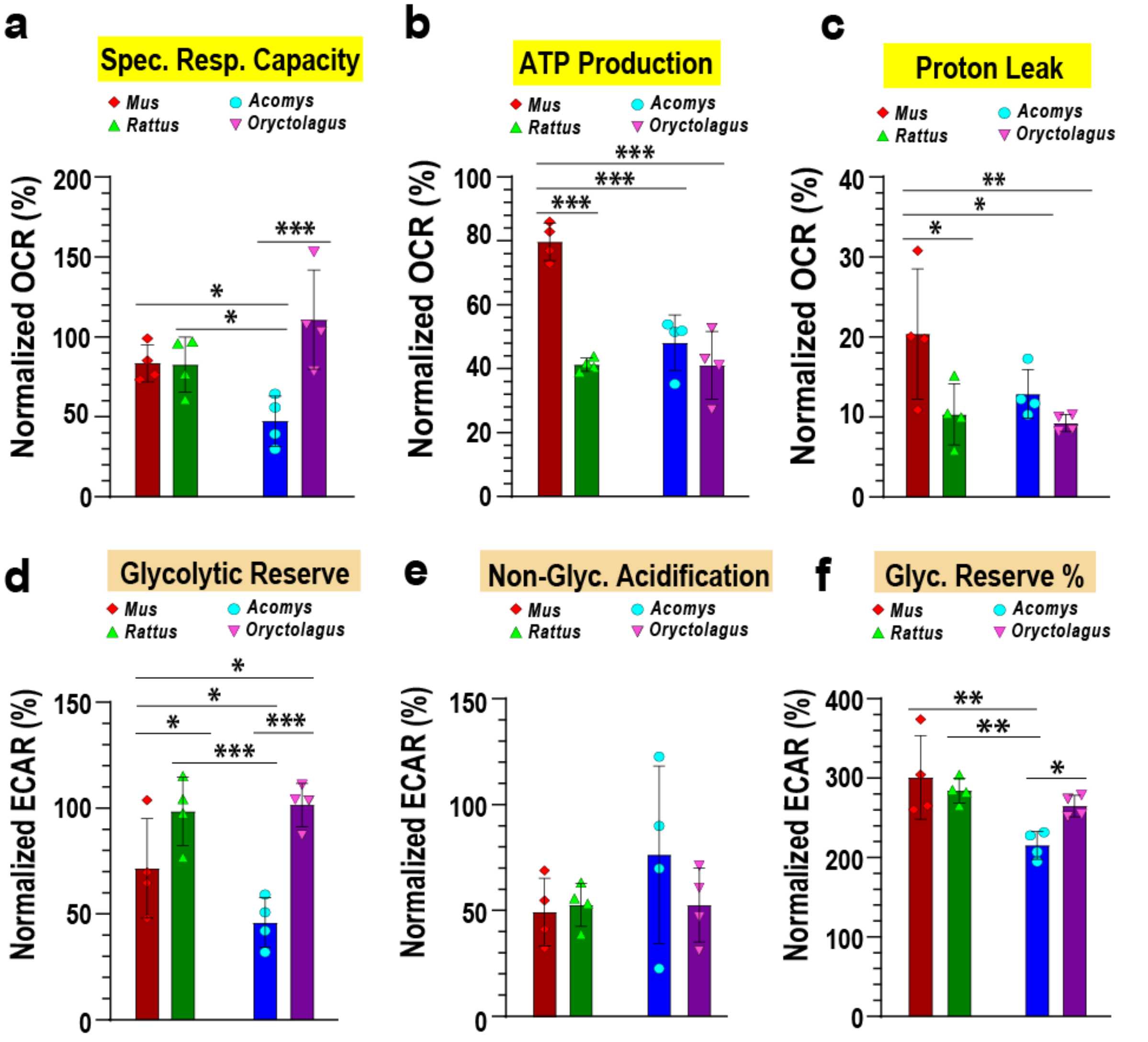

Supplementary Figure 1. Glycolytic and mitochondrial respiration parameters in *Mus*, *Acomys Rattus* and *Oryctolagus* fibroblasts. Oxidative Phosphorylation and glycolytic parameters determined in all four species from the MST-mitochondrial stress tests **(a-c)** and GST-Glycolytic stress tests **(d-f)** run on Seahorse XFe96 analyzer using primary ear pinna fibroblasts (continued from Fig. 1). While *Mus* showed the highest levels of ATP production (ANOVA, F=24.0693, p<0.0001) and proton leak (ANOVA, F=4.4067, p=0.0262), rabbit fibroblasts interestingly shared higher specific respiratory capacity with *Mus* and *Rattus*, all three significantly higher than *Acomys* (ANOVA, F=6.5691, p=0.0071). Glycolytic reserve levels were distinctively higher in *Rattus* and *Oryctolagus* compared to *Acomys* and *Mus* (ANOVA, F=10.3251, p=0.0012). All ECAR parameters across all four species were normalized to the average basal glycolytic rate in *Mus*. All OCR parameters were also normalized to the average basal respiration rate in *Mus*. Individual data points represent biological replicates (n=4) for each species. Post-hoc pairwise comparisons were carried out using the students' t-test. (* p<0.05, ** p<0.001, *** p<0.0001).
